# S240. DETERMINANT FACTORS OF REAL-WORLD FUNCTIONING IN SCHIZOPHRENIA

**DOI:** 10.1093/schbul/sby018.1027

**Published:** 2018-04-01

**Authors:** Leticia González-Blanco, M Paz Garcia-Portilla, Leticia Garcia-Alvarez, Lorena De La Fuente-Tomas, Pilar Saiz-Martinez, Celso Iglesias, Ana Coto, Julio Bobes

**Affiliations:** University of Oviedo

## Abstract

**Background:**

Negative, cognitive and depressive symptoms, as well as physical comorbidities, have a great impact on the real-world functioning in patients with schizophrenia (SZ) (1, 2, 3). However, not all the studies have employed accurate psychometric instruments to assess these symptoms, nor have all these factors been studied simultaneously.

The aim of the current study is to analyze the determinants of functionality in SZ measured by the Personal and Social Performance (PSP) scale, and considering not exclusively psychopathological and cognitive variables, but also aspects related to physical health and inflammation.

**Methods:**

Sample: 73 outpatients with SZ, duration of illness ≤10 years, under stable maintenance treatment [mean age (31.7 ± 6.5), males (61.6%)].

Clinical variables: PANSS, CGI-Severity, Clinical Assessment Interview of Negative Symptoms (CAINS) -Motivation/Pleasure (MAP) & Expression (EXP) domains-, Brief Negative Symptom Scale (BNSS), Calgary Depression Scale (CDS), MATRICS Consensus Cognitive Battery (MCCB), PSP.

Biological variables: Glucose, cholesterol, LDL, HDL, triglycerides, TSH, prolactin, insulin, uric acid, alkaline phosphatase (APh), C-reactive protein (CRP), TNF-α, interleukin(IL)-6, IL-2, IL-1β, IL-1RA, homocysteine, HT (% hemolysis), lipid peroxidation (LPO), catalase.

Pearson correlations were performed to select variables significantly related to PSP scores which were later included in stepwise multiple linear regression analyses. Age, sex, education, smoking, alcohol use, BMI, antipsychotic equivalent doses and other confounding factors were considered.

**Results:**

Final model for PSP total score (R2=0.778, F=45.564, p<0.001) identified that CGI-Severity (β= -0.279), PANSS-NM (negative Marder Factor) (β = -0.218), Asociality subscale of BNSS (β= -0.383) and IL-2 (β= -0.269) were significant predictors.

Predicting variables included in regression models for specific PSP domains:

- Self-care (R2=0.661, F=22.947, p<0.001): PANSS-NM (β=0.458), Avolition subscale of BNSS (β=0.248), IL-2 (β=0.221), APh (β=0.201).

- Useful activities (R2=0.563, F=42.473, p<0.001): CGI-S (β=0.245), Avolition (β=0.557).

- Social relationships (R2=0.731, F=56.998, p<0.001): Asociality (β=0.578), PANSS-GP (β=0.276), CAINS-EXP (β=0.178).

- Aggresive behaviour (R2=0.335, F=16.892, p<0.001): PANSS-P (β=0.408), CDS (β=0.273).

**Discussion:**

1.Negative symptoms are the most important determinants of a deficit in the real-world functioning in SZ, especially “asociality.”

2.“Apathy” has a negative impact on self-care and useful activities domains.

3.Proinflammatory cytokine IL-2 marks poor functionality.

**References:**

1. Harvey (2014). Assessing disability in schizophrenia: tools and contributors. J Clin Psychiatry.

2. Strassnig et al. (2015). Determinants of different aspects of everyday outcome in schizophrenia: The roles of negative symptoms, cognition, and functional capacity. Schizophr Res.

3. Menendez-Miranda et al. (2015). Predictive factors of functional capacity and real-world functioning in patients with schizophrenia. Eur Psychiatry.

